# Assessment of the Environmental Impact of Solid Oil Materials Based on Pyrolysis Oil

**DOI:** 10.3390/ma16175847

**Published:** 2023-08-26

**Authors:** Anita Staroń, Magda Kijania-Kontak, Mariusz Dziadas, Marcin Banach

**Affiliations:** 1 Department of Engineering and Chemical Technology, Cracow University of Technology, 24 Warszawska St., 31-155 Cracow, Poland; 2 Department of Civil Engineering, Cracow University of Technology, 24 Warszawska St., 31-155 Cracow, Poland; 3 Faculty of Chemistry, University of Wrocław, 14F. Joliot-Curie St., 50-383 Wrocław, Poland

**Keywords:** pyrolysis oil, oil block, oil binder, phytotoxicity, mechanical strength, environmental assessment

## Abstract

One method of managing used car tires is decomposition by thermochemical conversion methods. By conducting the process at temperatures of 450–750 °C, three fractions are obtained from tires: oil, gas, and solid. The liquid product of the pyrolysis of used car tires is pyrolysis oil, which consists of aromatic, polyaromatic, and aliphatic hydrocarbons. Unconventional building materials were obtained from tire pyrolysis oil and the environmental impact was evaluated. Blocks made from pyrolysis oil showed mechanical strength of up to about 1700 N. No heavy metals or polycyclic aromatic hydrocarbons, which were found in the crude heavy-PO fraction, were detected in the filtrates after incubation of the block obtained from the heavy-PO fraction at 240 °C. The highest inhibition of *Sorghum saccharatum* shoot (74.4%) and root (57.5%) growth was observed for solid materials from the medium-PO fraction obtained at 240 °C. The most favorable values of the parameters for the process of obtaining blocks based on post-PO were an annealing temperature of 180 °C, time of 20 h, and mass ratio of catalyst to catalyzed oil of 0.045.

## 1. Introduction

It is estimated that around 1 billion used tires are retired annually worldwide, half of which are abandoned or illegally dumped. This amount is projected to rise to 1.2 billion units by 2030 [1,2]. Due to the variable composition of tires and their high calorific value, they have the potential to be used in the power industry or for the production of energy materials [3]. Decomposition of waste tires by thermochemical conversion methods involves heating shredded or whole tires at 400–750 °C without oxygen [4]. The pyrolysis process can be carried out in several ways. The main distinctions in the literature are gas–solid systems, liquid–solid systems, and conducting the process under reduced pressure [5,6]. In vacuum pyrolysis, on the other hand, the reactor is heated with molten salts. The process temperature is lower, at 480–520 °C, and the pressure is 20 kPa [7]. In addition, by performing simulated distillation, better fractional separation results are obtained than with atmospheric distillation. Thus, it is possible to achieve a final boiling point of about 600 °C, which indicates the presence of heavy hydrocarbon compounds in the pyrolysis oil [6]. A number of new studies are being carried out in the field of pyrolysis affecting the quality of the products obtained [8,9].

In the process of tire pyrolysis, three fractions are obtained: oil, gas, and solids. Further processing allows the acquisition of valuable raw materials such as 10–30% pyrolysis gas, 38–55% pyrolysis oil, and 33–38% carbonizate [10]. The quantities of products obtained depend strictly on the parameters for conducting the pyrolysis process, including temperature, pressure, time, and heating rate [11]. It is estimated that from one ton of waste tires in this process, 350 liters of synthetic light oil can be obtained, as well as technical carbon black and metal waste, which after melting is reused. The substrate used in the process, i.e., used tires, is most often shredded beforehand and stripped of metal parts [12].

Pyrolysis oil (PO) consists of more than 100 chemical compounds [13,14]. The main components are aromatic hydrocarbons (such as benzene, toluene, and xylenes), polyaromatic hydrocarbons (naphthalene), and aliphatic hydrocarbons. This indicates the presence of mainly saturated and unsaturated aliphatic, aromatic, and cyclic compounds with 7–20 carbon atoms. There may also be hydrocarbon derivatives containing oxygen, sulfur, or nitrogen in the oil. In addition, resinous substances may be present in the PO. PO is a dark and thick liquid with an intense odor. The light fraction, with a boiling point below 160 °C, accounts for 20% by weight of the resulting oil; the medium fraction, with a boiling point of 160–204 °C, accounts for 6.8%; the heavy fraction, with a boiling point of 204–350 °C, accounts for 30.7%; and the residue, or compounds with a boiling point above 350 °C, accounts for 42.5% [15,16]. The total PO is a mixture of all the fractions and is called averaged oil. Separate fractions are not a desirable product because heavy oil does not burn completely, while light oil does not have favorable starting properties as a fuel [17,18,19]. PO has a high calorific value of up to 44 MJ/kg, making it an alternative to conventional liquid fuel [20]. However, due to impurities, it can only be used as a fuel for industrial heating equipment. The use of the oil as a softener for rubber mixtures is also possible, despite it containing aromatic hydrocarbons [21]. The problem with using PO is that it contains sulfur, which comes from the vulcanization of rubber. Its content can be as high as 1%, making the oil unsuitable for direct use as an engine fuel. A correlation of higher concentrations of sulfur compounds in PO with an increasing temperature of the pyrolysis process has been observed [6]. Liquid products resulting from the pyrolysis of tires are classified as hazardous waste. The problem that arises during the management of waste PO is the presence of polycyclic aromatic hydrocarbons (PAHs) and heavy metals, which have adverse effects on human life as well as the environment [22,23].

Due to the large amount of tire PO generated, new ways of managing it are being sought. Attempts have been made to create unconventional building materials based on waste oils. The literature has mainly focused on vegetable waste oils from the thermal processing of food [24,25,26]. No data were found on the acquisition of oil blocks based on post-pyrolysis oil; therefore, an attempt was made in this work to produce solid composite materials (oil blocks) based on pyrolysis oil.

Using oil blocks made from oil from the pyrolysis of automobile tires as a building material, there is a risk that harmful components of PO will enter the environment and accumulate in plants and living organisms. It should be investigated whether the solid oil matrix produced from the oil from the pyrolysis of automobile tires can be safely used in an environment where it will come into contact with soil and water. Due to the presence of a number of compounds in the PO that are potentially hazardous to the environment, this paper investigates the acquisition of unconventional building materials from PO from the tire pyrolysis process and evaluates their impact on the environment.

The aim of the research was to investigate the possibility of obtaining solid oil materials based on oil derived from the pyrolysis of tires, and to assess their impact on the natural environment, which will determine the potential application of these materials.

## 2. Experimental

The study used post-pyrolysis oils (Pos) from the pyrolysis of automobile tires—light oil fraction (PO-L), medium oil fraction (PO-M), and heavy oil fraction (PO-H). The catalyst used in the process of obtaining solid oil materials was concentrated sulfuric(VI) acid with a purity of p.p.a. Sand with a fraction size of 0.1–0.5 mm was used as a filler.

The density of post-POs was determined in accordance with ASTM D4052 [27]. Kinetic and dynamic viscosities were determined at 20 and 100 °C using viscosity meter Stabinger Viscometer SVM 3001 (Anton Paar GmbH, Graz, Austria) in accordance with ASTM D 7052 [28]. Water content was determined by a coulometric method in accordance with ASTM D 6304C [29] using a Aquamax KF coulometric titrator (GR Scientific, Silsoe, UK). In order to observe the changes occurring in the different fractions of PO during their heating, thermal analysis of the oils was performed using SDT 650 apparatus (TA Instruments New Castle, USA) in the temperature range of 25–1000 °C and a temperature increment of 10 °C/min. Determination of metal content was carried out by energy-dispersive X-ray fluorescence using a Epsilon1 spectrometer (Malvern PANalytical, Malvern, UK). Calibration of the instrument was carried out on VHG-certified standards. Determination of PAHs in POs was carried out by LC-ESI-MS/MS. The molecular structure of the three fractions of PO, catalyzed PO (with a catalyst-to-catalyzed-oil mass ratio of: 0.045, 0.09, and 0.135), and solids was studied by Fourier-transform infrared spectroscopy using a Nicolet iS5 FT-IR spectrometer (Thermo Fisher Scientific, Waltham, MA, USA) in the wavelength range of 500–4000 cm^−1^.

### 2.1. Preparation of Oil Blocks Based on Post-Pyrolysis Oils and Their Characteristics

To obtain oil blocks, PO was mixed with an acid catalyst and homogenized for 10 min, then sand was added and mixed for another 5 min. The sample was transferred to an aluminum mold and annealed in a laboratory chamber oven.

Variable parameters of the process were annealing temperature (210–240 °C), annealing time (12–20 h), and mass ratio of catalyst to catalyzed PO (0.045, 0.09, and 0.135). The amount of catalyzed oil was constant at 25% by weight of sand. The process parameters for obtaining oil blocks are shown in Table 1.

Surface analysis of the oil blocks was performed using a Hitachi TM-3000 scanning electron microscope (Hitachi High-Technologies Corporation, Tokyo, Japan) equipped with an EDS X-ray microanalyzer. Strength testing was carried out using a Zwick-Roell Z600 testing machine (Zwick Roell Group, Ulm, Germany) (initial force of 25 N, test speed of 1 kN/min). In order to determine the leachability of heavy metals and PAHs from the blocks obtained from the POs, the blocks were crushed into approximately 1 cm pieces, and 19 g was weighed from each sample. The material was poured with deionized water at a mass ratio of 1:15 and shaken. After 1 week, the solid phase was separated from the filtrate. Determination of heavy metals (copper, iron, magnesium, tin, and zinc) in the filtrate was carried out by atomic absorption spectrometry using a Perkin-Elmer 370 spectrophotometer (Perkin Elmer, Waltham, MA, USA). Determination of PAHs in the filtrate was carried out by LC-ESI-MS/MS. The absorbability of the oil blocks was determined by measuring the weight of the blocks 24 h and 7 days after immersing them in an air-dry state in deionized water, then the ratio of the weight of water absorbed by the material to the weight of the dry material was determined.

### 2.2. Phytotoxicity and Water Pollution from Oil Blocks

In order to determine the effects of PO-based materials on plants, a phytotoxicity test was conducted using Tigret’s Phytotoxkit tests (according to ISO 18763 [30]). Plants were exposed to the filtrate from a 7-day incubation of the PO blocks in deionized water. The content of pigments and enzymes in plants exposed to oil block incubation filtrate was determined. The plant used in the studies was the monocotyledonous *Sorghum saccharatum*. A measure of 50 g of soil (Tigret, Warsaw, Poland) was weighed in containers and 100 sorghum seeds were placed at equal distances in each (5 rows of 20 seeds each). The seeds were watered with seepage or deionized water (control trial) and covered with foil to ensure better germination conditions. On the eighth day, the foil was removed, while the seeps were replaced with deionized water.

Catalase activity was measured by the UV spectrophotometric method, which involves monitoring the change in absorbance of 240 nm at high concentrations of hydrogen peroxide solution [31]. Determination of peroxidase in *S. saccharatum* leaves was carried out according to the methodology in [32]; absorbance of the solution was measured at 420 nm.

The content of assimilatory pigments (chlorophyll and carotenoids) was determined at 470, 647, and 664 nm after sample preparation according to the procedure proposed by Oren et al. [33]. Contamination of water in contact with the oil blocks was assessed on the basis of inhibition of ingestion by living organisms using the acute toxicity microbiotest—Rapidtoxkit F (Tigret, Warsaw, Poland) [34]. This standardized bioassay conforms to ISO Standard 14380 [35]. The crustacean *Thamnocephalus platyurus* was used for the study.

### 2.3. Statistical Analysis

Approximation profiles were created to determine the values of independent parameters to obtain the most favorable estimated values of the outcome factor. The approximated values of the output factor for combinations of input factor values were converted to a usability scale. The usability values of a dependent variable can vary from 0.0 (undesirable) to 1.0 (highly desirable) [36]. Statistical analysis was conducted in version 10 of STATISTICA by StatSoft^®^ [37].

## 3. Results and Discussion

### 3.1. Pyrolytic Oil

Physicochemical parameters of PO fractions, such as kinetic and dynamic viscosity, water content, and presence of heavy metals and PAHs, are shown in Table 2.

**Figure 1:**
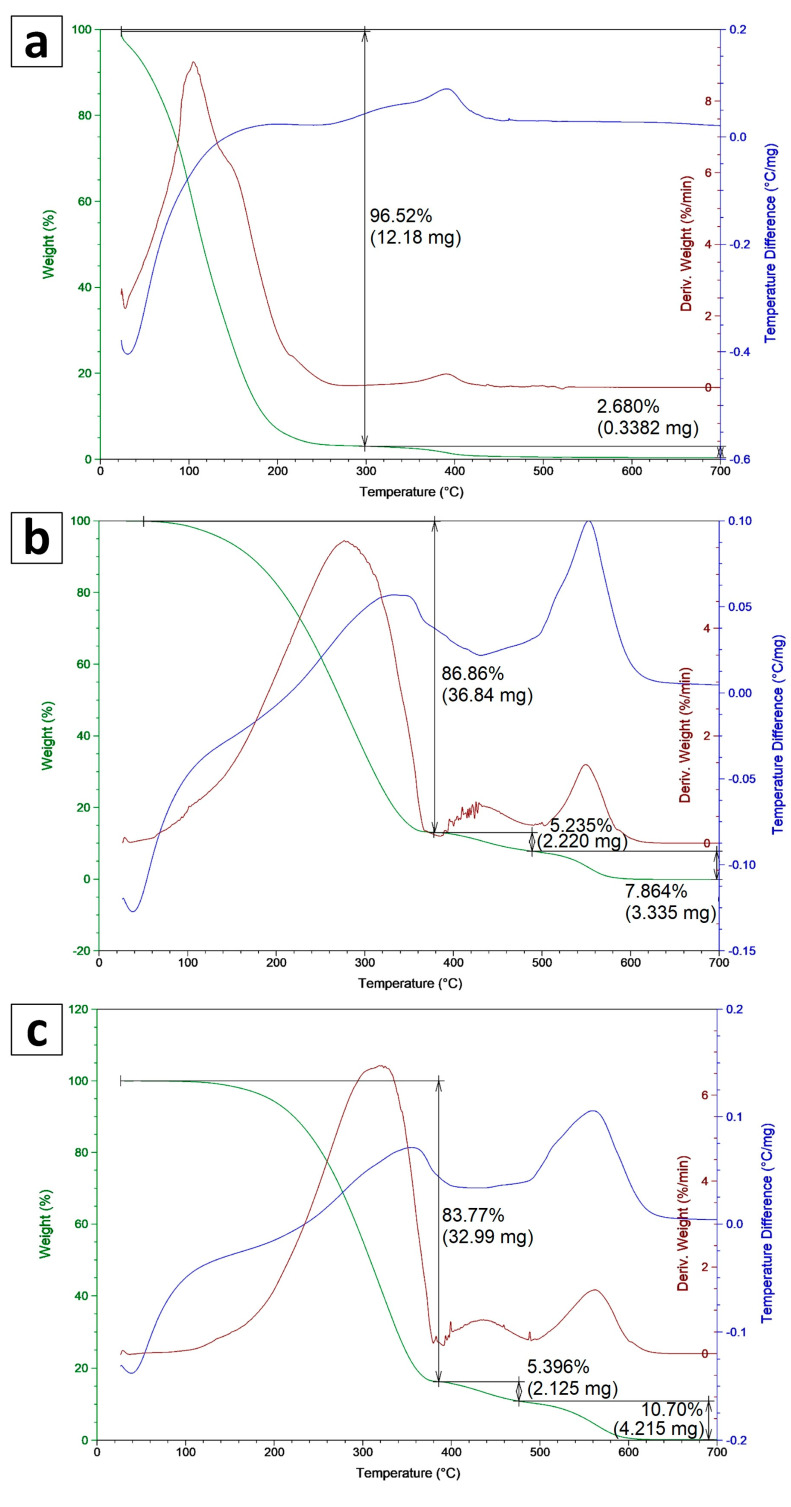
TG, DTG, and DTA graphs for pyrolytic oil: (**a**) light, (**b**) medium, and (**c**) heavy fractions.

The results of TGA-DTA thermal analysis for fractions of PO are shown in Figure 1. For PO-L, the highest mass loss (96.5%) occurred in the temperature range of 25–300 °C. For PO-L and PO-H, mass degradation occurred gradually and the highest mass loss (86.9% and 83.8%, respectively) occurred in the temperature range of 50–400 °C. In the temperature range of 400–500 °C, the mass loss was about 5% for PO-L and PO-H, while in the range of 500–600 °C, it was 7.9% and 10.7%, respectively. Above 600 °C, the sample mass stabilized.

The DTG curve for PO-L shows peaks corresponding to two-stage degradation. The first stage reached a maximum degradation rate of 9%/min at about 110 °C. In the second stage, on the other hand, the rate was very low at 0.5%/min, corresponding to the degradation of a small mass of oil at about 390 °C. For PO-M and PO-H, there are three peaks corresponding to the three stages of oil degradation. The maximum degradation rate of 5.5%/min for medium oil occurred at 280 °C, while for heavy oil it was 6.5%/min at 340 °C. The subsequent degradation steps of the samples were identical for both oil samples and their values were 0.75%/min at 440 °C and 1.5%/min at 550 °C, respectively. At lower temperatures, lower hydrocarbons are released, most of which are found in light oil, while higher hydrocarbons with lower volatility are found in heavier oil fractions.

**Figure 2:**
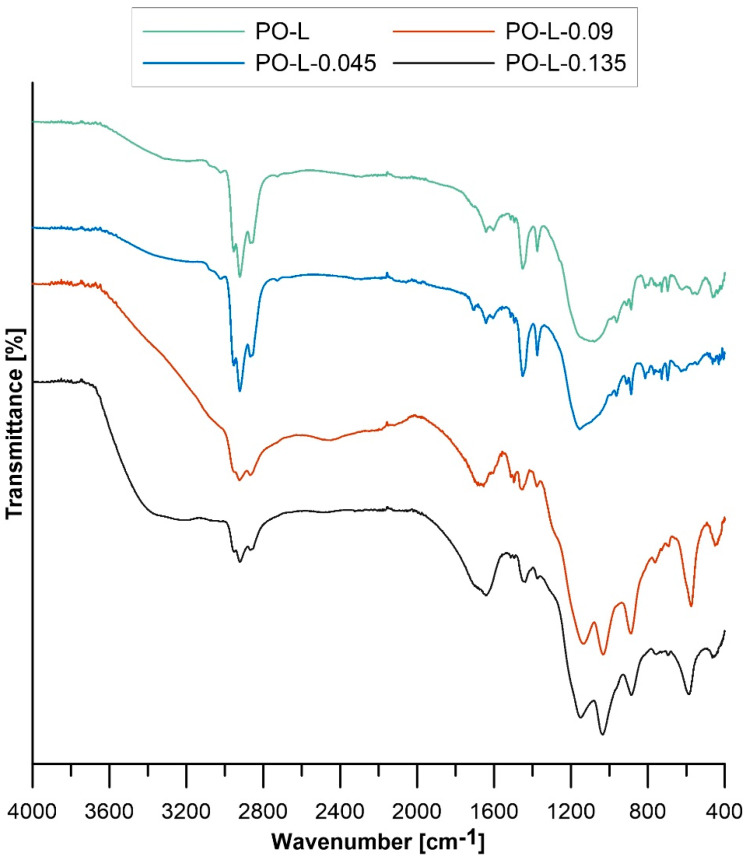
FT-IR spectra for pyrolytic light oil and pyrolytic light oil catalyzed with catalyst-to-catalyzed-oil weight ratios of 0.045, 0.09, and 0.135.

The results of FT-IR spectrometric analysis for PO-L along with the catalyzed oils with a catalyst-to-catalyzed-oil mass ratio of 0.09, 0.045, and 0.135 are shown in Figure 2.

For each sample, there are sharp peaks at wave numbers 2922 and 2866 cm^−1^ corresponding to C–H bond stretching vibrations in aliphatic and aromatic compounds. The peaks occurring at 1451 and 1375 cm^−1^ correspond to scissor bending vibrations of the C–H bond (in alkanes) and symmetric bending vibrations of the C–H bond (methylene group) [38,39]. The peak appearing for each sample with a wave number of 1641 cm^−1^ can be attributed to stretching vibrations of the C–C bond present in alkanes. In turn, the peaks present in the 1150–1000 cm^−1^ region correspond to bending vibrations of the C–H bond of aromatic compounds. The peaks appearing at 887 and 814 cm^−1^ in the case of PO-L correspond to stretching vibrations of the C=C bond and bending vibrations outside the C–H bond plane. They testify to the presence of di- and tri-substituted alkenes. In the case of catalyzed PO-L with a catalyst-to-catalyzed-oil mass ratio of 0.135, the peaks corresponding to these compounds occur at a wave number of 875 cm^−1^ [39,40]. The peaks at 728 and 697 cm^−1^ can be attributed to single-substituted aromatic compounds. In addition, the peaks appearing in the range 1328–1035 cm^−1^ are due to the presence of sulfoxides and sulphonic derivatives [41]. For PO to which more sulfuric acid was added, a sharpening of the peaks in the 1150–1000 cm^−1^ range is observed. The presented FT-IR spectrum for pyrolytic light oil shows similarity to the FT-IR spectrum of waste tires [38].

### 3.2. Oil Blocks Based on Post-Pyrolysis Oils

**Figure 3:**
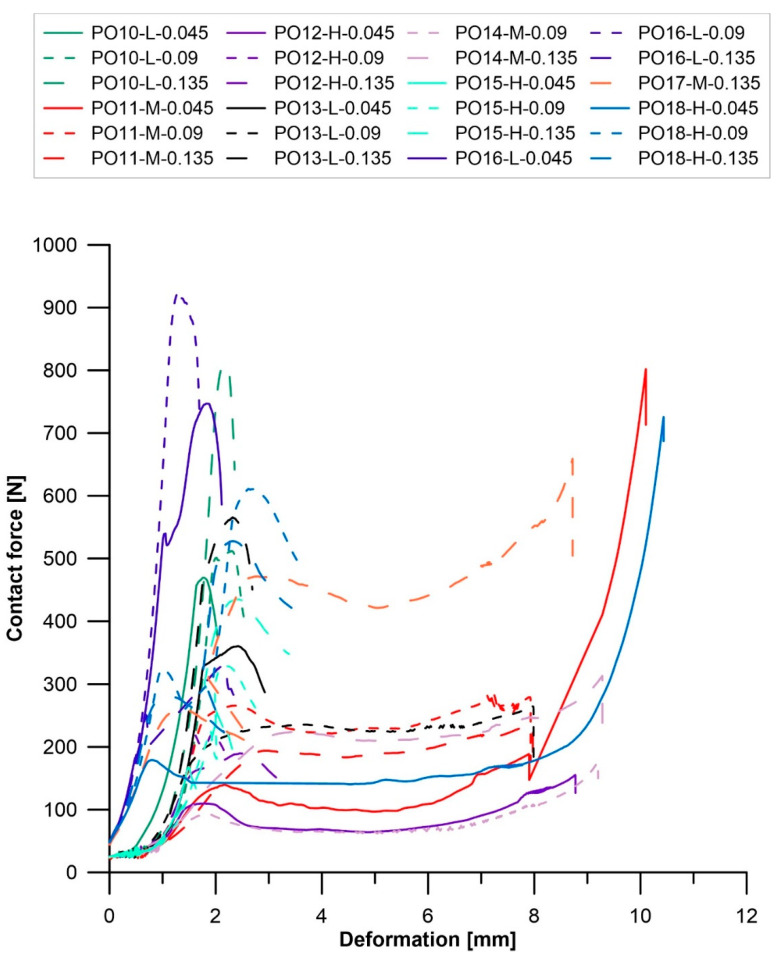
Mechanical strength of solid oil materials obtained at 210 °C.

**Figure 4:**
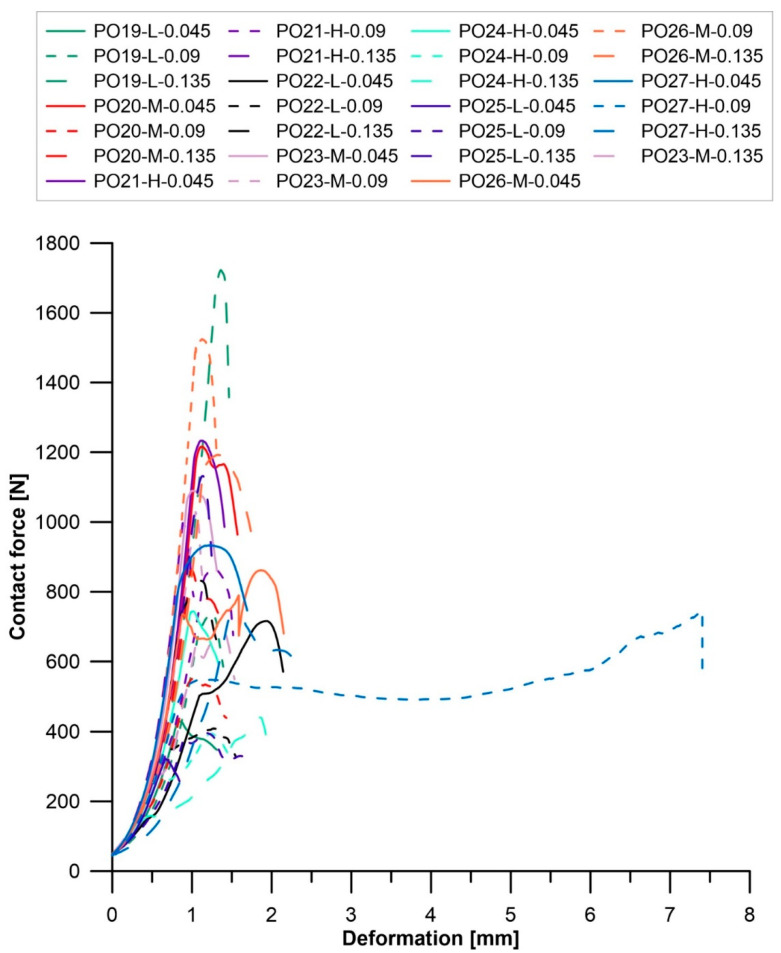
Mechanical strength of solid oil materials obtained at 240 °C.

The results for the mechanical strength of the samples are presented by plotting the relationship of the applied force (N) against the strain of the samples (mm). The mechanical strength of solid oil materials obtained at 210 and 240 °C is shown in Figure 3 and Figure 4, respectively.

For the samples obtained at 210 °C, the most favorable ratio of applied standard force to strain was obtained for two samples: PO10-L-0.135 and PO16-L-0.09. Both of them were prepared from PO-L. A significant number of samples had very low mechanical strength (less than 800 N), these were: PO11-M-0.045, PO11-M-0.135, PO12-H-0.045, PO14-M-0.09, and PO14-M-0.135. Most of these samples were obtained from PO-M. Thus, it can be concluded that regardless of the ratio of catalyst to catalyzed oil, a temperature of 210 °C is not suitable for obtaining solid materials from PO-M. Oil blocks obtained at 240 °C had the highest strength parameters compared to the strength of materials obtained at lower temperatures. The highest strength was characterized by 14 samples, including: PO19-L-0.135, PO20-M-0.045, PO21-H-0.045, PO23-M-0.045, PO23-M-0.09, PO23-M-0.135, PO26-M-0.09, and PO26-M-0.135. In the case of the samples annealed at 240 °C, no relationship was observed between the strength of the material and the fraction of PO from which the oil block was formed. Moreover, no relationship was observed between the strength of the blocks and the ratio of catalyst to catalyzed oil. Based on the results obtained from the dependence of the applied standard force on the strain of the specimen, it can be concluded that the higher the annealing temperature of the oil blocks, the higher the strength of the material. For comparison, blocks based on waste cooking oil obtained at 170 °C had a compressive strength of about 34 MPa and stiffness of almost 20.16 MPa, while blocks obtained at 200 °C had the lowest values of these parameters due to high porosity [25]. Oil blocks obtained by the polymerization of waste cooking oil in the presence of an acid catalyst differed in mechanical strength depending on the process parameters in which they were obtained. The highest compressive strength of about 2600 N was characterized by blocks containing sulfuric(VI) acid in a weight ratio of 0.03 to WCO, and the highest annealing temperature was 220 °C [42].

The specimens that had the lowest deformation and at the same time had a standard force value of not less than 800 N were selected for further testing and are shown in Table 3.

The strength values of PO blocks are comparable to those of solid materials obtained from waste cooking oil (WCO) [42]. The weight of oil blocks exposed to an aqueous environment increased over time, which was due to the penetration of water into the pores of the material. Sample PO21-H-0.135, which was made from the heavy fraction of PO, had the highest absorbency. On the other hand, the lowest absorbability was recorded for sample PO19-L-0.135, obtained from the light fraction of PO. The soaking value for this sample after 7 days was lower than after 24 h, which may be due to the rapid degradation of the sample in an aqueous environment, thus reducing its weight. Lower soaking values were also obtained for samples annealed at lower temperatures (210 °C): PO10-L-0.135 and PO16-L-0.09. The samples were made with PO-L. Analyzing the results, it can be seen that as the density of the PO fraction increases, the ability of the oil block to absorb water through the material increases.

Obtaining a solid material from PO occurs through a polymerization reaction, which results in the saturation of unsaturated bonds of the compounds contained in the oil. Monomers under the influence of temperature and a catalyst form dimers. These, in turn, combine with subsequent mers to form complex chains of high molecular weight. Compounds that polymerize in PO include methylhexadiene, octene, methyloctene, and pentadecane [43].

PO also undergoes an esterification reaction. Compounds that have a hydroxyl functional group (e.g., phenol, dimethylphenol) and a carboxyl group (e.g., benzoic acid), as a result of heating, form dimers. Under the influence of a catalyst which is a Bronsted acid (e.g., sulfonic acid or sulfuric(VI) acid), the compounds in the PO undergo a polyesterification reaction, resulting in a solid material [44].

In both cases, polymerization and esterification, these reactions occur under the influence of appropriate process conditions, such as temperature and the presence of catalysts. These conditions are crucial for the effective conversion of pyrolytic oil into a stable solid material.

The mentioned polymerization and esterification reactions are just two of many possible chemical processes that can occur during the transformation of pyrolytic oil into a solid material. There are many other reactions and mechanisms that can also play a significant role in the formation of stable compounds with higher molecular weight. Among them are condensation reactions, which lead to the merging of smaller units and the creation of larger molecules. Additionally, oxidation reactions have the potential to transform the compounds present in pyrolytic oil, forming more complex and stable compounds with a greater molecular weight. In the presence of different types of monomers or chemical compounds, copolymerization reactions can take place, resulting in the formation of stable copolymers with an increased molecular weight. Furthermore, cross-linking reactions can also play a significant role in the creation of a polymer network, enhancing the stability and molecular weight of the material. The investigation of these reactions and mechanisms is crucial for a better understanding of the processes involved in the creation of stable compounds with a higher molecular weight in solid oil materials [45,46].

**Figure 5:**
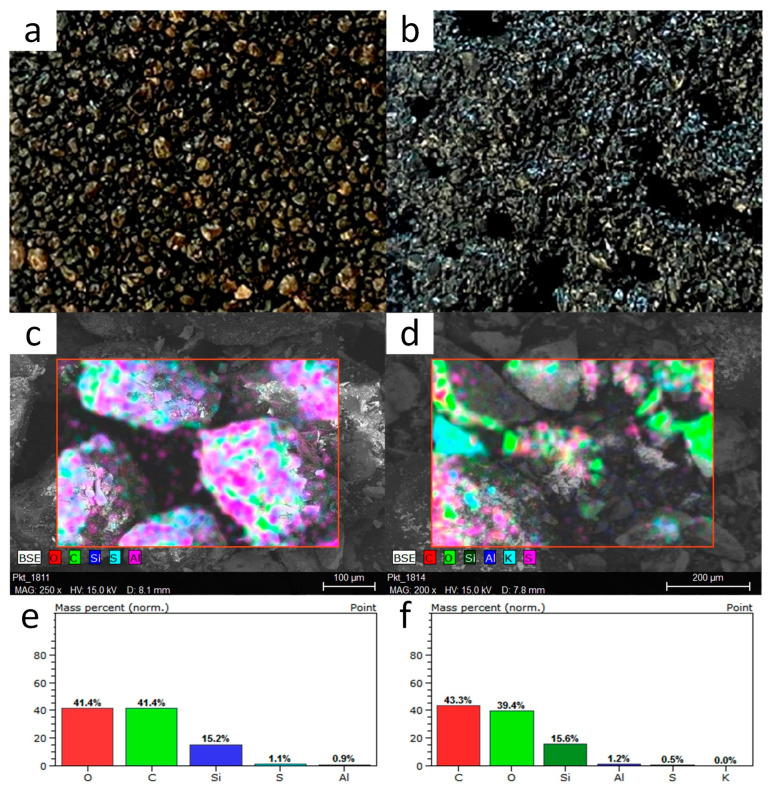
Photographs of oil blocks: (**a**) PO16-L-0.09, (**b**) PO24-H-0.135; SEM microphotographs: (**c**) PO16-L-0.09, (**d**) PO24-H-0.135; and EDS analysis: (**e**) PO16-L-0.09, (**f**) PO24-H-0.135.

The surface structure of the selected oil blocks is shown in Figure 5. The blocks formed based on PO have a similar structure. The presence of pores, which promote the accumulation of water in the structure of the blocks, is noted, which may affect the saturation of the material. Sand grains permanently fused in the oil matrix can also be seen. Sharp edges were formed where the material was fractured. All the samples obtained had a dark color.

EDS X-ray analysis of the solids showed that they consisted mainly of carbon, oxygen, and silicon. The difference in silicon concentration between the samples is due to the point-like nature of the analysis (silicon comes from sand added to a mixture of PO and sulfuric acid), hence the small amounts of aluminum, sulfur, and calcium atoms.

**Figure 6:**
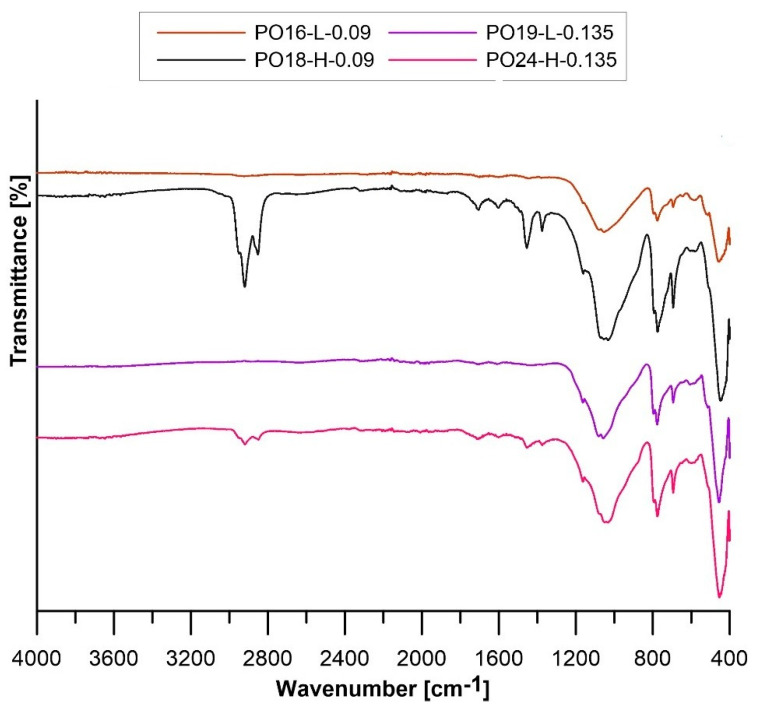
FT-IR spectra of solid samples.

Figure 6 shows the results of FT-IR analysis of selected six solid material samples: PO16-L-0.09, PO18-H-0.09, PO19-L-0.135, and PO23-H-0.045.

For samples PO16-L-0.09 and PO19-L-0.135, three characteristic peaks are visible. The first is located at a wave number of 1055 cm^−1^ and corresponds to bending vibrations of C–H bonds present in aromatic compounds. The peaks present at 777 and 694 cm^−1^ originate from the bending vibrations of the C–H bonds of single-substituent aromatic compounds [38,39,40]. For samples PO18-H-0.09 and PO24-H-0.135, there are distinct peaks at 2942, 2918, and 2852 cm^−1^ corresponding to stretching vibrations of C–H bonds. Another distinctive peak from bending vibrations of scissor C–H bonds is found at 1455 cm^−1^. The peak at 1705 cm^−1^ comes from stretching vibrations of C=O bonds, characteristic of aldehydes and ketones [47]. Bending vibrations of C–H bonds present in aromatic compounds occur at 1033 cm^−1^. The peaks located at 755 and 694 cm^−1^ come from single-substituted aromatic compounds. In addition, a peak at 1602 cm^−1^ corresponding to stretching vibrations of the C=C bond found in alkenes was noticed in sample PO18-H-0.09 [48]. The presence of this bond in the test sample may be indicative of poor polymerization of the oil in the oil matrix [40].

Molecular structure analysis of the analyzed oil blocks showed similarity for all samples except PO18-H-0.09 and PO24-H-0.135. The apparent difference in the wave number range 3000–2700 cm^−1^ may be due to the polymerization reaction, which did not occur correctly in these cases. This can also be evidenced by the material strength results, which were unfavorable for the aforementioned samples.

Table 4 compares the content of heavy metals and selected PAHs in the POs and in the filtrate after incubation of the oil blocks per gram of oil block.

No copper, which showed the highest concentration in PO-L, was found in the filtrate obtained after incubation of the oil blocks in deionized water. This indicates the binding of copper in the matrix structure of the oil block. The concentration of other heavy metals in the filtrate was much lower than in the individual fractions of the post-PO. The highest iron concentrations were obtained for the filtrates from the incubation of samples PO25-L-0.135, PO16-L-0.09, PO19-L-0.135, and PO22-L-0.135. These values were above 10 mg/L and each of these samples was made from PO-L, in which the iron concentration was the highest. In contrast, the lowest iron concentration was obtained for sample PO21-H-0.045 made from PO-H. The iron content of the filtrates is also affected by the amount of catalyst used; the lower the ratio of catalyst to catalyzed oil, the lower the iron concentration in the filtrate. In British EQS, the limitation on discharges into the environment of iron is 1 mg/L. This means that only the filtrate from the incubation of sample PO21-H-0.045 did not exceed the limit on iron concentration in water [49].

As for the concentration of magnesium in the filtrates, the highest concentrations were obtained for samples PO10-L-0.135 and PO19-L-0.135, made from the light fraction of PO. On the other hand, the highest concentration of tin was found in the filtrates from samples made from the medium fraction of PO (PO26-M-0.045 and PO26-M-0.09) due to the highest concentration of tin in this fraction of oil. In addition, a high concentration of tin was found in the filtrate from sample PO21-H-0.09 made from the heavy fraction of PO, despite the lowest concentration of tin in PO-H. Zinc was found in the highest concentration in the heavy fraction of the PO, meaning it was also found in the highest concentration in the filtrate of samples made from this oil (PO21-H-0.135). In addition, comparing the values for samples PO21-H-0.045, PO21-H-0.09, and PO21-H-0.135, it can be seen that increasing the ratio of catalyst to catalyzed oil increased the amount of zinc leached from the oil block. On the other hand, the filtrates obtained from the medium fraction of PO (PO23-M-0.045, PO26-M-0.09, and PO26-M-0.135) did not show the presence of zinc.

The content of PAHs in the filtrates after incubation of oil blocks per gram of sample was significantly lower than for the oil used to obtain 1 g of oil block. This indicates that a significant amount of PAHs are permanently bound in the oil matrix. However, for most samples, leaching of such amounts of PAHs was observed, which may have harmful effects on the environment. For sample PO27-H-0.045, no leaching of PAHs was observed, which may indicate the most favorable parameters for conducting the process of obtaining oil blocks based on post-POs, which are: annealing temperature of 240 °C, annealing time of 20 h, and $m_{H_{2}{SO}_{4}}$/$m_{olej\;skat.}$= 0.045.

#### 3.2.1. Phytotoxicity of Oil Blocks Based on Post-Pyrolysis Oils

**Figure 7:**
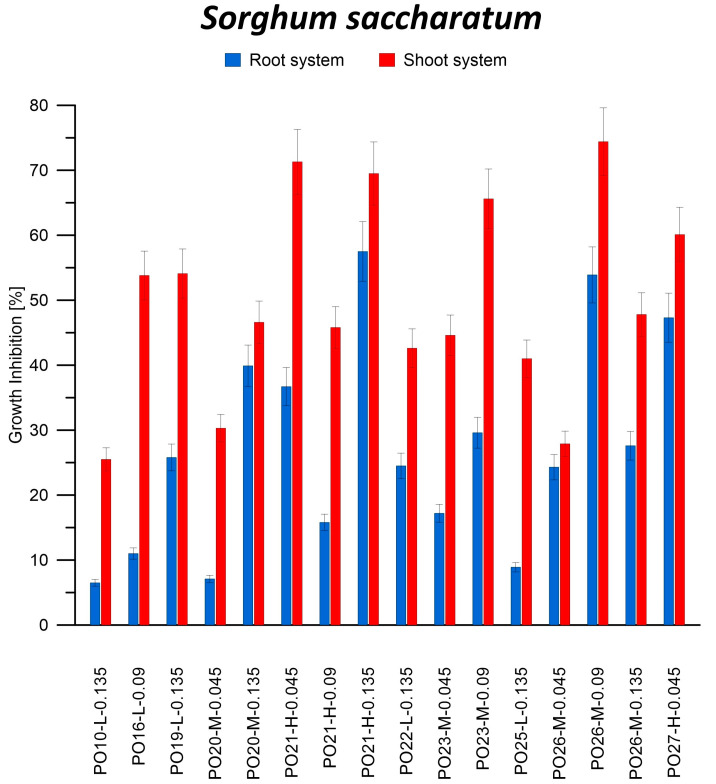
Inhibition of shoot and root growth of *Sorghum saccharatum* on medium from reference soil soaked in filtrate after incubation of oil blocks in deionized water.

**Figure 8:**
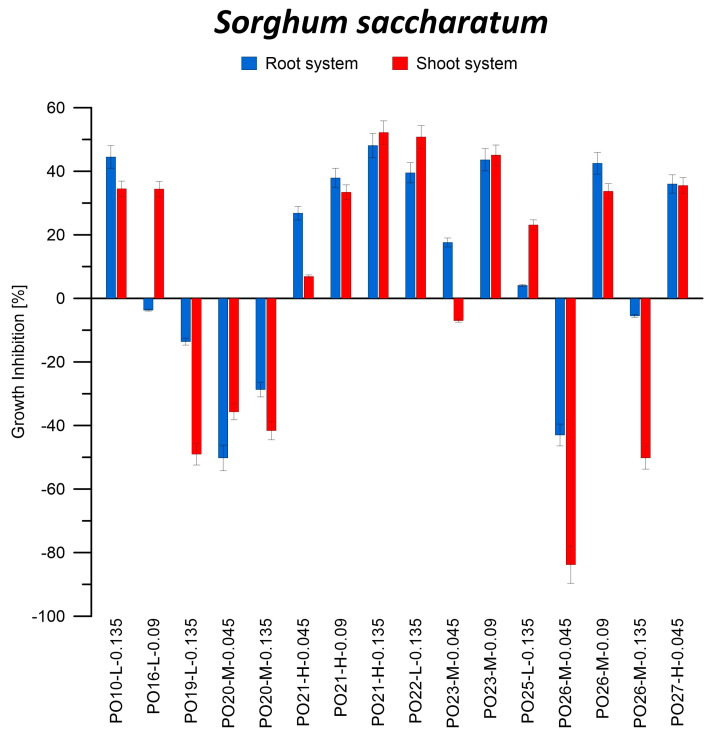
Inhibition of shoot and root growth of *Sorghum saccharatum* on medium from reference sponge soaked in filtrate after incubation of oil blocks in deionized water.

The highest percentage of inhibition of *S. saccharatum* shoot growth in the soil medium (74.4%) was obtained for the filtrate from sample PO26-M-0.09. On the other hand, for the roots of this plant, the highest inhibition (57.5%) was obtained using the filtrate from sample PO21-H-0.135. Both samples were annealed at 240 °C (Figure 7). When using sponge as a substrate, the highest inhibition of *S. saccharatum* growth (both shoots and roots; 52.2% and 48.1%, respectively) was obtained for the filtrate of sample PO21-H-0.135 (Figure 8).

Positive inhibition values were obtained for all seeds in contact with the leakage after a 7-day incubation of oily blocks on the soil medium. This indicates a decrease in the growth of both shoots and roots of *S. saccharatum* in contact with substances leached from oily blocks. Zang et al. also reported similar findings for both aboveground (−22.9%) and belowground (−8.4%) growth under the influence of microplastics [50]. In the case of microplastics, it is likely that although they cannot penetrate the root cuticle of the tested radish plants, they still negatively affect plant growth. In the investigated composites, both leached components and micro-particles can exert an influence.

In the case of seeds germinating on spongy substrate, inhibition of growth of shoots and roots of *S. saccharum* was mainly observed. It can therefore be concluded that the components of the leakage had a detrimental effect on the plant, disturbing its proper development, and the interaction of soil components and substances leached from PO blocks increases the negative impact on plant development. Based on the presented results, it can be concluded that PO blocks have a strong phytotoxic effect. These blocks are therefore not safe for the environment, unlike WCO blocks. In a study in which oil blocks obtained from WCO were incubated in soil saturated with deionized water and rainwater, an increase of up to 75% and 135.1%, respectively, in the above-ground part of the plant was observed compared to the control sample [42].

Numerous studies have also demonstrated that the presence of plastics in the soil can have varying effects on plant growth and morphological characteristics [51]. For instance, non-biodegradable plastics such as petroleum-based polypropylene did not affect the germination of higher plant seeds but did influence their growth. The observed effects ranged from growth inhibition to stimulation. Dicotyledonous plants exhibited particular sensitivity to plastics, as evidenced by changes in root and stem length, which occurred for each type of plastic tested. Exposure to polyhydroxybutyrate and polylactide-based plastics resulted in up to a 22% inhibition of plant root growth [52]. Each PO block used in our study had a different composition or degree of polymerization, leading to varying impacts on plants growth. Similar findings were reported by Qi et al., who observed that different types of plastic had varying effects on wheat, influencing both aboveground and underground development to different extents [53].

This negative effect of PO blocks on plant development can be explained by the leachability of organic substances containing PAHs from the resulting materials. Similar observations on the harmful effects of PAHs on plant growth have been reported in the literature [54]. These compounds can be transported across cell membranes, causing them damage and increasing their permeability, which consequently leads to the inhibition of plant growth [55]. In addition, PAHs have been shown to interfere with the process of photosynthesis, which first leads to inhibition of plant growth and then plant death [56,57]. Daresta et al. [58] observed a decrease in the growth of tomato seedlings and an increase in the level of ROS in plant cells exposed to atmospheric PAHs. Similar conclusions were reported by Pašková et al. [59], who carried out research using mustard, barley, and beans under the influence of three PAHs and their N-heterocyclic derivatives. Desalme et al. [60] presented the negative effect of phenanthrene on the example of clover. The lower growth rate of the plant is therefore due to the toxic effect of one or more components that leached from the PO block.

In addition, no effect of the annealing temperature of the oil blocks, the amount of catalyst used, or the type of PO fraction from which the samples were made was found on the growth inhibition of shoots and roots of *S. saccharatum*. The activity of catalase, peroxidase, assimilation dyes, and dry weight in *S. saccharatum* leaves is shown in Table 5.

The highest concentrations of chlorophyll a and b in *S. saccharatum* plants were recorded for samples made from PO-M. The highest chlorophyll concentration (412.0 mg/g fresh weight) was achieved for sample PO26-M-0.045. At the same time, the concentration of heavy metals was the lowest for samples made with PO-M, so it can be inferred that heavy metals affect the chlorophyll content of plants. Comparing the catalyst content in the oil blocks to the chlorophyll content in the plants, it can be seen that as the ratio of catalyst to catalyzed oil increased (at the same time as the pH of the filtrate decreased), the amount of chlorophyll in the plants decreased. In the case of carotenoids, analogous relationships to those observed for chlorophyll were achieved.

Chlorophyll and carotenoids are vital pigments that play essential roles in the survival and functioning of plant organisms. Consequently, changes in chlorophyll content serve as indicators of oxidative stress in plants caused by various abiotic factors, such as salinity, drought, unfavorable temperatures, or the presence of harmful environmental chemicals. The reduction in carotenoid content in plants grown in contact with substances leached from PO blocks suggests potential damage to photosystems and disruptions in their functionality. Moreover, an increase in carotenoid content relative to total chlorophyll content indicates a certain level of plant activation prior to the onset of this stress. The interplay between carotenoid synthesis and catabolism plays a critical role in determining carotenoid accumulation [61]. A particularly noticeable disproportion in the content of assimilation pigment is observed in a plant grown in soil containing PO blocks: PO26-M-0.135 and PO21-H-0.045. It can be hypothesized that the low content of the acid catalyst contributes to the poor polymerization of the post-pyrolysis oil, and consequently the substances from the PO blocks are released into the environment. The high acid content of the catalyst causes the transfer of its components to the environment. In both cases, plants are stressed.

The catalase activity in plants exposed to substances leached from oil blocks decreased over time. In the range of 0–60 s, the highest catalase activity (335.3 μmol H_2_O_2_/g) was obtained for sample PO22-L-0.135. This value differs significantly from the others, and from that obtained for the control sample, which may indicate the presence of compounds that promote catalase activity in the filtrate. In the following minutes, catalase activity decreased with a decreasing concentration of H_2_O_2_ in the sample until it reached zero for samples PO20-M-0.045 and PO23-M-0.09. On the other hand, for peroxidase, activity increased with an increasing catalyst/catalyzed oil ratio and decreasing pH value of the filtrate. The highest value (9.2 mol/min·mL) was obtained for sample PO26-M-0.135. Peroxidase activity was significantly lower for the filtrate than for the control sample (30.8 mol/min·mL).

The findings regarding antioxidant enzyme activity in crop plants grown in soil containing PO blocks are consistent with existing literature. Peroxidases are widely distributed in microorganisms, plants, and animals, with predominant localization in the cell wall. These enzymes serve as key regulators of plant growth and development, playing crucial roles in various cellular processes such as cell wall construction, reinforcement, and lignification. The elimination of two major enzymatic systems responsible for hydrogen peroxide removal and protection against peroxidative cell damage is an essential mechanism for preserving biological membranes [62,63]. The activity of catalase and peroxidase in cell walls is controlled by the effectiveness of the antioxidative peroxidase enzyme system [64]. The metabolic function of peroxidase remains ambiguous due to its participation in numerous catalytic reactions and the presence of many isoenzymes [65,66]. The activity of peroxidase is closely associated with the quality of plant-based products, particularly in relation to flavor, in both raw and processed foods. In numerous instances, peroxidase activity has been correlated with fruit ripening and enzymatic browning processes, often in conjunction with the activity of polyphenol oxidase [67,68].

Exposure of plants to PAHs causes oxidative stress, which causes disturbances in their vital functions, e.g., in the process of photosynthesis. This causes yellowing of the leaves and disturbances in the production of assimilation dyes, including a decrease in the content of chlorophyll, as well as modifications in the structure of the chloroplast [60,69]. Jin et al. [70] described the influence of phenanthrene on the activity of photosystem II. This hydrocarbon blocks photosynthetic electron transport and photochemical reactions (e.g., redox, reversible bleaching), negatively affecting chlorophyll pigments [71,72].

#### 3.2.2. Contamination of the Water in Contact with the Oil Blocks Based on Post-Pyrolysis Oils

**Figure 9:**
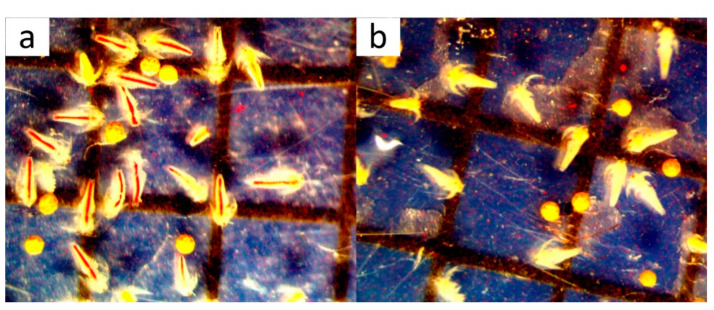
*Thamnocephalus platyurus* larvae in: (**a**) control sample and (**b**) filtrate in contact with oil block PO21-H-0.09.

Contamination of the water that came into contact with the oil blocks was assessed based on the food-taking ability of *Thamnocephalus platyurus* larvae. Figure 9 shows *T. platyurus* larvae after contact with substances leached from the oil block and the control.

In the control sample, using standard medium, staining of the digestive tracts of *T. platyurus* larvae can be observed. The appearance of unhatched cysts and early-stage larvae that are incapable of ingesting food can also be seen. To compile the results, early-stage larvae and unhatched cysts were not included in the total number of organisms. The proportion of *T. platyurus* larvae with stained digestive tracts was 87%. When the larvae were incubated in the tested filtrates, it can be seen that food intake was inhibited in each sample: 0% of *T. platyurus* larvae had stained digestive tracts. The result of the rapid water contamination assessment test indicates the presence of toxins, causing complete inhibition of food intake by *T. platyurus* larvae. Thus, it can be concluded that the components leached from the PO-based blocks are dangerous contaminants that threaten living organisms.

#### 3.2.3. Advantages and Disadvantages of Solid Oil Materials Based on Pyrolysis Oil

The use of solid oil-based materials brings a number of benefits, making them an attractive option in various sectors, including the construction industry. Firstly, the production of these materials is characterized by relatively simple technologies, and the utilization of oil obtained from the pyrolysis process of tires as a raw material contributes to cost reduction.

The high strength of solid oil-based materials makes them suitable for a variety of construction applications. Additionally, they exhibit low water absorption, which positively affects their durability and resistance to degradation. By modifying process parameters, the time required to obtain the final product can be shortened, which is significant in terms of production efficiency.

However, it is important to remember certain drawbacks associated with the production of solid oil-based materials. The high energy intensity associated with the curing process can lead to increased energy consumption. Furthermore, the production scale of these materials may be limited due to the available furnace surface area and the availability of raw materials, which is dependent on the geopolitical situation, posing challenges to the stability of production.

Another disadvantage is the variable chemical composition of solid materials based on oil derived from tire pyrolysis. The presence of heavy metals and other undesirable compounds can affect the quality and safety of the final materials. Thus, in order to fully consider the use of solid materials based on pyrolysis oil in the construction industry, it is necessary to optimize the process of obtaining them in such a way that the resulting material does not have a negative impact on the environment.

### 3.3. Statistical Analysis of the Results

**Figure 10:**
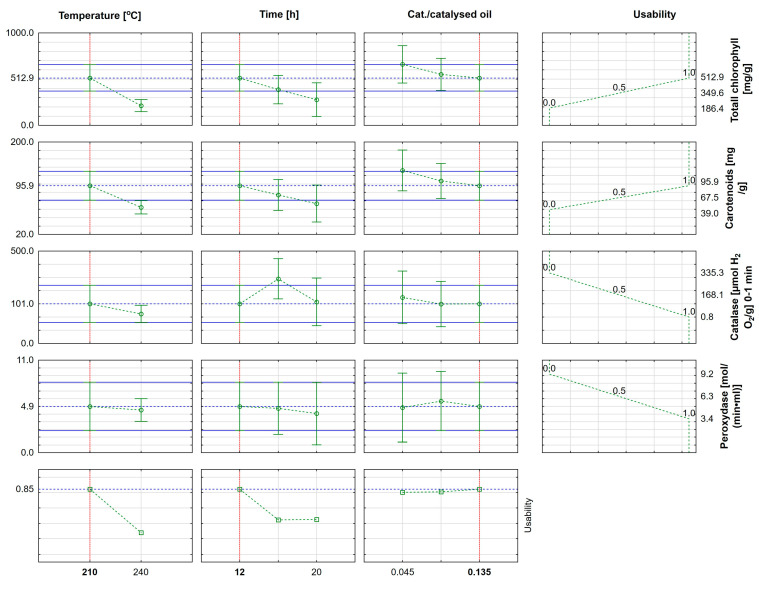
Approximation profile and utility function for obtaining PO-based oil blocks in terms of chlorophyll a and b, carotenoid, and peroxidase content in *Sorghum saccharatum* leaves.

Approximation profile and utility function for obtaining PO-based oil blocks in terms of chlorophyll a and b, carotenoid, and peroxidase content in *Sorghum saccharatum* leaves are shown in Figure 10.

The most favorable parameters for running the process were an annealing temperature of 210 °C, annealing time of 12 h, and catalyst-to-catalyzed-oil ratio equal to 0.135. Usability was equal to 85%.

By changing the process parameters for obtaining oil blocks based on post-pyrolysis oil, it is possible to produce materials that have the least invasive impact on the environment. It is important that future research of oil blocks be based on the principles of sustainable development presented and using advanced tools for assessing sustainability, including technical and economic ones, such as life cycle assessment and energy assessment [73].

## 4. Conclusions and Future Perspectives

The oil blocks obtained based on pyrolysis oils showed high mechanical strength (up to 1720 N). The highest mechanical strength was achieved for oil blocks annealed at 240 °C. The absorbability of the oil blocks increases with an increase in the density of the PO used. The concentration of heavy metals and PAHs in the filtrates was much lower than in the POs used to obtain the oil blocks. Most PAHs leached from oil blocks obtained at 180 °C. No leaching of PAHs was observed in the case of block PO27-H-0.045 (obtained at 240 °C). The highest *S. saccharatum* shoot and root growth inhibition was obtained for the reference soil substrate, amounting to 74.4% (PO26-M-0.09) and 57.5% (PO21-M-0.135), respectively. Both samples were annealed at 240 °C from the PO medium fraction. As the ratio of catalyst to catalyzed oil increases, the content of assimilation pigments in *S. saccharatum* decreases. Peroxidase activity in *S. saccharatum* exposed to oil blocks increases as the catalyst/catalyzed oil ratio increases and the pH value decreases. Food intake by *T. platyurus* larvae was 100% inhibited, indicating contamination of water in contact with oil blocks. The environmental assessment carried out proves that it is possible to modify the parameters for obtaining oil blocks so that leaching of heavy metals and PAHs in contact with the environment is as low as possible. With the applied process parameters, the obtained oil blocks containing post-pyrolysis oil as a binder adversely affect the growth of plants and living organisms, which is a limitation in terms of their use, in particular for construction purposes, where they would be exposed to contact with external factors. The practical implications of this study include the use of waste material, but at the same time the possibility of introducing harmful substances into the environment.

## Figures and Tables

**Table 1:** Process parameters for obtaining oil blocks containing 25% catalyzed pyrolytic oil of light (PO-L), medium (PO-M), and heavy (PO-H) fractions.

Sample	H_2_SO_4_/PO_cat_ (m/m)	PO (g)	H_2_SO_4_ (g)	Time (h)	Temp. (°C)	Sand (g)
PO10-L-0.045	0.045	7.16	0.34	12	210	30
PO10-L-0.09	0.09	6.83	0.68			
PO10-L-0.135	0.135	6.49	1.01			
PO11-M-0.045	0.045	7.16	0.34			
PO11-M-0.09	0.09	6.83	0.68			
PO11-M-0.135	0.135	6.49	1.01			
PO12-H-0.045	0.045	7.16	0.34			
PO12-H-0.09	0.09	6.83	0.68			
PO12-H-0.135	0.135	6.49	1.01			
PO13-L-0.045	0.045	7.16	0.34	16		
PO13-L-0.09	0.09	6.83	0.68			
PO13-L-0.135	0.135	6.49	1.01			
PO14-M-0.045	0.045	7.16	0.34			
PO14-M-0.09	0.09	6.83	0.68			
PO14-M-0.135	0.135	6.49	1.01			
PO15-H-0.045	0.045	7.16	0.34			
PO15-H-0.09	0.09	6.83	0.68			
PO15-H-0.135	0.135	6.49	1.01			
PO16-L-0.045	0.045	7.16	0.34	20		
PO16-L-0.09	0.09	6.83	0.68			
PO16-L-0.135	0.135	6.49	1.01			
PO17-M-0.045	0.045	7.16	0.34			
PO17-M-0.09	0.09	6.83	0.68			
PO17-M-0.135	0.135	6.49	1.01			
PO18-H-0.045	0.045	7.16	0.34			
PO18-H-0.09	0.09	6.83	0.68			
PO18-H-0.135	0.135	6.49	1.01			
PO19-L-0.045	0.045	7.16	0.34	12	240	
PO19-L-0.09	0.09	6.83	0.68			
PO19-L-0.135	0.135	6.49	1.01			
PO20-M-0.045	0.045	7.16	0.34			
PO20-M-0.09	0.09	6.83	0.68			
PO20-M-0.135	0.135	6.49	1.01			
PO21-H-0.045	0.045	7.16	0.34			
PO21-H-0.09	0.09	6.83	0.68			
PO21-H-0.135	0.135	6.49	1.01			
PO22-L-0.045	0.045	7.16	0.34	16		
PO22-L-0.09	0.09	6.83	0.68			
PO22-L-0.135	0.135	6.49	1.01			
PO23-M-0.045	0.045	7.16	0.34			
PO23-M-0.09	0.09	6.83	0.68			
PO23-M-0.135	0.135	6.49	1.01			
PO24-H-0.045	0.045	7.16	0.34			
PO24-H-0.09	0.09	6.83	0.68			
PO24-H-0.135	0.135	6.49	1.01			
PO25-L-0.045	0.045	7.16	0.34	20		
PO25-L-0.09	0.09	6.83	0.68			
PO25-L-0.135	0.135	6.49	1.01			
PO26-M-0.045	0.045	7.16	0.34			
PO26-M-0.09	0.09	6.83	0.68			
PO26-M-0.135	0.135	6.49	1.01			
PO27-H-0.045	0.045	7.16	0.34			
PO27-H-0.09	0.09	6.83	0.68			
PO27-H-0.135	0.135	6.49	1.01			

**Table 2:** Physical and chemical properties of light, medium, and heavy fractions of oil from the pyrolysis of automobile tires.

Parameter		PO-L	PO-M	PO-H
Water (ppm)		569	674	788
Kinetic viscosity at 20 °C (cSt)		1.96	98.9	1213.6
Kinetic viscosity at 100 °C (cSt)		0.72	4,24	10.11
Dynamic viscosity at 20 °C (cSt)		1.71	95.01	1187.7
Dynamic viscosity at 100 °C (cSt)		0.59	3.84	9.35
Heavy metals (ppm)	K	5	15	4
	Na	5	<1	<1
	Ba	2	3	2
	Ca	40	25	29
	Cr	1	<1	<1
	Cu	42	<1	<1
	Fe	387	113	83
	Mg	151	4	15
	Sn	41	70	16
	Zn	4	12	54
Polycyclic aromatic hydrocarbons (μg/L)	naphthalene	565	258	46
	acenaphthylene	13	16	4
	acenaphthene	90	141	25
	fluorene	84	105	43
	phenanthrene	79	183	117
	anthracene	27	62	43
	fluoranthene	47	161	149
	pyrene	84	284	293
	benzo(a)anthracene	8	45	56
	chrysene	9	62	78
	benzo(b)fluoranthene	7	37	46
	benzo(k)fluoranthene	3	7	10
	benzo(a)pyrene	7	32	57
	dibenzo(a,h)anthracene	6	20	25
	benzo(g,h,i)perylene	5	11	12
	indeno(1,2,3-cd)pyrene	9	54	74

**Table 3:** Solid materials with a mechanical strength of more than 800 N and their absorbability after 24 h and 7 days.

Sample	Mechanical Strength (N)	Absorbability—24 h (%)	Absorbability—7 Days (%)
PO10-L-0.135	810	2.63	4.91
PO16-L-0.09	923	3.91	4.38
PO19-L-0.135	1720	3.55	3.39
PO20-M-0.045	1220	5.16	7.30
PO20-M-0.135	863	6.02	7.98
PO21-H-0.045	1230	4.12	6.75
PO21-H-0.09	866	7.31	6.64
PO21-H-0.135	997	8.05	9.50
PO22-L-0.135	832	5.19	5.19
PO23-M-0.045	1090	6.16	6.79
PO23-M-0.09	1030	5.03	6.76
PO25-L-0.135	1130	5.81	7.88
PO26-M-0.045	862	3.55	5.09
PO26-M-0.09	1520	3.14	6.01
PO26-M-0.135	1190	4.05	5.24
PO27-H-0.045	933	3.61	5.41

**Table 4:** Comparison of heavy metals and polycyclic aromatic hydrocarbons contained in oil and filtrate per gram of oil block.

Sample	Sn (mg)		Zn (mg)		Mg (mg)		Fe (mg)		Pyrene (μg)		Fluoranthene (μg)		Anthracene (μg)		Phenanthrene (μg)		Fluoranthene (μg)		Acenaphthene (μg)		Naphthalene (μg)	
	Filt.	Oil	Filt.	Oil	Filt.	Oil	Filt.	Oil	Filt.	Oil	Filt.	Oil	Filt.	Oil	Filt.	Oil	Filt.	Oil	Filt.	Oil	Filt.	Oil
PO10-L-0.135	0.01	0.11	<0.001	0.01	0.06	0.39	0.13	1.00	0.001	0.76	0.001	0.39	0.009	0.11	0.002	0.30	0.001	0.11	0	0.06	<0.001	0.12
PO16-L-0.09	0.02	0.11	<0.001	0.01	0.05	0.41	0.21	1.06	0.001	0.80	0.001	0.41	0.009	0.12	0.002	0.32	0.001	0.12	0.002	0.07	<0.001	0.13
PO19-L-0.135	0.02	0.11	<0.001	0.01	0.06	0.39	0.17	1.00	0.001	0.76	0.001	0.39	0	0.11	0.002	0.30	0.001	0.11	0.002	0.06	<0.001	0.12
PO20-M-0.045	0.02	0.12	<0.001	0.03	0.01	0.01	0.05	0.32	0	0.81	0	0.46	0	0.18	0.002	0.52	0.001	0.30	0	0.40	<0.001	0.74
PO20-M-0.135	0.03	0.18	<0.001	0.03	0.02	0.01	0.09	0.29	0	0.74	0	0.42	0	0.16	0.002	0.47	0	0.27	0	0.36	<0.001	0.67
PO21-H-0.045	0.02	0.05	<0.001	0.15	0.01	0.04	0.01	0.24	0.001	0.84	0.001	0.43	0	0.12	0.002	0.33	0.001	0.12	0.002	0.07	<0.001	0.13
PO21-H-0.09	0.04	0.04	<0.001	0.15	0.02	0.04	0.04	0.23	0.001	0.80	0	0.41	0	0.12	0.002	0.32	0.001	0.12	0	0.07	<0.001	0.13
PO21-H-0.135	0.02	0.04	<0.001	0.14	0.03	0.04	0.13	0.22	0	0.76	0	0.39	0	0.11	0.002	0.30	0.001	0.11	0.002	0.06	<0.001	0.12
PO22-L-0.135	0.02	0.11	<0.001	0.01	0.04	0.39	0.17	1.00	0	0.76	0.001	0.39	0	0.11	0.002	0.30	0.001	0.11	0	0.06	<0.001	0.12
PO23-M-0.045	0.04	0.20	<0.001	0.03	0.01	0.01	0.03	0.32	0.001	0.24	0.001	0.13	0	0.08	0.002	0.23	0.001	0.24	0.002	0.26	<0.001	1.62
PO23-M-0.09	0.03	0.19	<0.001	0.03	0.03	0.01	0.06	0.31	0	0.78	0	0.44	0	0.17	0	0.50	0.001	0.29	0	0.38	<0.001	0.70
PO25-L-0.135	0.03	0.18	<0.001	0.01	0.06	0.39	0.24	1.00	0.001	0.76	0.001	0.39	0	0.11	0.002	0.30	0.001	0.11	0.002	0.06	<0.001	0.12
PO26-M-0.045	0.04	0.20	<0.001	0.03	0.01	0.01	0.03	0.32	0.001	0.24	0.001	0.13	0	0.08	0.002	0.23	0.001	0.24	0	0.26	<0.001	1.62
PO26-M-0.09	0.04	0.19	<0.001	0.03	0.02	0.01	0.08	0.31	0	0.78	0	0.44	0	0.17	0.002	0.50	0.001	0.29	0	0.38	<0.001	0.70
PO26-M-0.135	0.02	0.18	<0.001	0.03	0.02	0.01	0.06	0.29	0.001	0.76	0.001	0.39	0	0.11	0.002	0.30	0.001	0.11	0	0.06	<0.001	0.12
PO27-H-0.045	0.01	0.05	<0.001	0.15	0.01	0.04	0.02	0.24	0	0.24	0	0.13	0	0.08	0	0.23	0	0.24	0	0.26	<0.001	1.62

**Table 5:** Activity of catalase, peroxidase, and assimilation dyes in *Sorghum saccharatum*.

Sample	Assimilation Dyes (mg/g Fresh Mass)				Catalase (μmol H_2_O_2_/g])				Peroxidase (moL/min·mL)
	Chlorophyll a	Chlorophyll b	Total Chlorophyll	Carotenoids	0–1 min	1–2 min	2–3 min	3–4 min	
CONTROL	348.8	102.1	450.8	97.3	40.8	18.8	12.7	2.8	30.8
PO10-L-0.135	363.8	149.1	512.9	95.9	-	-	-	-	5.0
PO16-L-0.09	215.9	67.5	283.4	54.0	57.1	22.4	7.0	3.2	3.4
PO19-L-0.135	157.4	52.1	209.5	43.3	7.2	1.1	3.9	3.0	4.0
PO20-M-0.045	283.3	108.3	391.6	88.1	130.0	17.2	1.6	0.0	4.7
PO20-M-0.135	175.5	58.4	233.9	49.7	0.8	7.7	1.1	5.8	4.8
PO21-H-0.045	146.9	62.6	209.5	39.0	51.2	2.7	1.3	5.7	4.0
PO21-H-0.09	139.7	46.6	186.4	42.1	9.1	3.0	11.9	11.4	5.0
PO21-H-0.135	170.7	68.2	238.9	52.2	2.2	4.6	10.6	9.8	4.9
PO22-L-0.135	150.5	51.3	201.8	47.1	335.3	33.0	22.9	18.0	7.4
PO23-M-0.045	248.5	77.8	326.3	69.8	134.3	32.1	1.7	19.1	5.1
PO23-M-0.09	206.4	74.4	280.7	62.4	181.2	43.8	9.2	0.0	5.2
PO25-L-0.135	228.5	69.4	297.8	67.8	13.8	2.5	7.2	18.3	5.7
PO26-M-0.045	302.1	110.0	412.0	90.0	27.6	7.0	1.6	9.2	6.1
PO26-M-0.09	222.9	66.7	289.6	60.4	51.6	13.0	11.0	16.0	8.6
PO26-M-0.135	138.7	58.8	197.5	39.6	58.6	17.8	1.9	7.4	9.2
PO27-H-0.045	174.4	58.5	232.9	47.5	16.6	9.8	6.3	3.0	3.4

## Data Availability

Data sharing not applicable.
